# Bactericidal Action of Shrimp Shell Chitooligosaccharide Conjugated with Epigallocatechin Gallate (COS-EGCG) against *Listeria monocytogenes*

**DOI:** 10.3390/foods12030634

**Published:** 2023-02-02

**Authors:** Jirayu Buatong, Ajay Mittal, Pimonsri Mittraparp-arthorn, Suriya Palamae, Jirakrit Saetang, Soottawat Benjakul

**Affiliations:** 1 International Center of Excellence in Seafood Science and Innovation, Faculty of Agro-Industry, Prince of Songkla University, Hat Yai 90110, Songkhla, Thailand; 2 Division of Biological Science, Faculty of Science, Prince of Songkla University, Hat Yai 90110, Songkhla, Thailand

**Keywords:** *Listeria monocytogenes*, chitooligosaccharide, polyphenol, conjugate, antimicrobial, bactericidal mechanism

## Abstract

The antibacterial effect of chitooligosaccharide conjugated with five different polyphenols, including catechin (COS-CAT), epigallocatechin gallate (COS-EGCG), gallic acid (COS-GAL), caffeic acid (COS-CAF), and ferulic acid (COS-FER), against *Listeria monocytogenes* was investigated. Among all the conjugates tested, COS-EGCG showed the highest inhibition toward *Listeria monocytogenes*, with a minimum inhibitory concentration (MIC) and minimum bactericidal concentration (MBC) of 1024 and 1024 µg/mL, respectively. The COS-EGCG conjugate also had a bactericidal effect on the environmental and clinical strains of *L. monocytogenes*. The low concentration of COS-EGCG conjugate augmented the formation of biofilm and the growth of *L. monocytogenes*. Nevertheless, the inhibition of biofilm formation and bacterial growth was achieved when treated with the COS-EGCG conjugate at 2 × MIC for 48 h. In addition, the COS-EGCG conjugate at 2 × MIC had the potential to inactivate the pre-biofilm, and it reduced the production of the extracellular polysaccharides of *L. monocytogenes*. The COS-EGCG conjugate at the MIC/4 effectively impeded the motility (the swimming and swarming) of *L. monocytogenes*, with an 85.7–94.3% inhibition, while 100% inhibition was achieved with the MIC. Based on scanning electron microscopic (SEM) images, cell wall damage with numerous pores on the cell surface was observed. Such cell distortion resulted in protein leakage. As a result, COS-EGCG could penetrate into the cell and bind with the DNA backbone. Therefore, the COS-EGCG conjugate could be further developed as a natural antimicrobial agent for inhibiting or controlling *L. monocytogenes*.

## 1. Introduction

*Listeria monocytogenes* is a facultative gram-positive bacterium and foodborne pathogen, in which the majority causes listeriosis. It is abundant in both environment and animal digestive tracts [1,2]. Raw and ready-to-eat (RTE) foods can be contaminated with soil or water during unhygienic processing [2,3]. Recently, *L. monocytogenes* has been documented to contaminate smoked and graved salmon, causing human death from listeriosis, with 17 cases in Germany [4]. The prevention of foodborne pathogens in food products is very crucial to save the consumer worldwide. According to the food laws of many countries, no *L. monocytogenes* must be detected in 25 g of RTE foods [5,6]. Chemical preservatives for food are often used to control foodborne pathogens and prolong products’ shelf life. Nonetheless, the use of chemical preservatives has a limitation since some chemicals are harmful to human health, such as allergic reactions, asthma, and neurological damage, and some of them are carcinogens [7,8]. In general, the hazard or risk of chemical preservatives depends on the dosage used and the frequency of intake. Those preservatives must, therefore, be applied, following the regulation or standard strictly.

Alternative natural preservatives have become crucial and challenging to use for killing or inhibiting the growth of foodborne pathogens. Some antimicrobial substances can be produced from waste generated during seafood processing. Among those, chitosan and chitooligosaccharides (COSs) from shrimp shells have gained increasing attention [9]. COSs from shrimp shells has a broad spectrum for antibacterial, antifungal, and antiviral activities [10,11,12,13]. Several mechanisms of COSs on antimicrobial activity have been proposed. Firstly, COS, having protonated amino groups, binds with the negatively charged bacterial cell wall. This phenomenon results in bacterial cell surface damage and nutrient leakage from the cells. Because of the low molecular weight of COSs (<10 kDa), with a low degree of polymerization (DP), COSs can penetrate through the bacterial cell and interact with DNA, thus inhibiting the DNA replication of the bacteria [14]. The mechanisms of EGCG on gram-positive and gram-negative bacteria have been intensively studied. EGCG has a strong efficacy in interacting with the head groups of the lipid bilayer of the bacterial cell wall via hydrogen bonds [15]. In addition, EGCG causes the pores of the outer membrane of *E. coli* and other gram-negative bacteria, thus releasing nutrients from the cells [16]. The binding of EGCG to bacterial protein, especially amino acids through hydrogen bonding, was predicted by molecular docking. The expression of the oligopeptide ABC transporter binding lipoprotein (Oppa) in *B. subtilis* was decreased in the presence of EGCG, resulting in the inhibition of the transport of oligopeptides [17]. Recently, COSs conjugated with several polyphenols, including catechin (CAT), epigallocatechin gallate (EGCG), gallic acid (GAL), caffeic acid (CAF), ferulic acid (FER), and proanthocyanidin, have been shown to possess antimicrobial, as well as antioxidative activities [18]. COSs conjugated with epigallocatechin gallate (COS-EGCG) have antibacterial activity toward both spoilage bacteria, including *Pseudomonas aeruginosa* (MBC/MIC, 128/64 µg/mL) and foodborne pathogens involving *Vibrio parahaemolyticus* (MBC/MIC, 128/64 µg/mL), *Escherichia coli* (MBC/MIC, 512/256 µg/mL), and *L. monocytogenes* (MBC/MIC, 512/128 µg/mL) [18].

However, the bactericidal mechanisms of the COS-EGCG conjugate toward *L. monocytogenes* have never been elucidated. Thus, this investigation aimed to examine the antimicrobial activity of COS conjugated with polyphenols against *L. monocytogenes* and to elucidate the possible bactericidal mechanism of the COS-EGCG conjugate toward *L. monocytogenes*.

## 2. Materials and Methods

### 2.1. Chemicals

Chitooligosaccharide (COS), with an average molecular weight (MW) of 0.7 kDa and a degree of polymerization (DP) of 2–8, and COS-polyphenol (PPN) conjugates (COS-PPN), was prepared following the method of Mittal et al. [18]. COS-PPN complexes were prepared by dissolving 1 g of COS in 100 mL of acetic acid (0.5%, *v*/*v*). Thereafter, COS was grafted with caffeic acid (CAF) (0.1% *w*/*w* of COS), catechin (CAT) (0.4% *w*/*w* of COS), epigallocatechin gallate (EGCG) (0.1% *w*/*w* of COS), ferulic acid (FER) (0.1% *w*/*w* of COS), and gallic acid (GAL) (0.2% *w*/*w* of COS). The degrees of conjugation were 19.60, 17.22, 29.32, 19.29, and 35.94%, respectively. Analytical grade chemicals were obtained from Sigma-Aldrich (St. Louis, MO, USA). All microbial media were purchased from HiMedia Laboratories (Mumbai, India).

### 2.2. Bacterial Strains and Culture Condition

Eleven *L. monocytogenes* strains were used. Those included the reference strain F2365 (gifted by the Food Safety Laboratory, Prince of Songkla University), five clinical strains (*L. monocytogenes* LM2, LM3, LM9, LM13, and LM14), and five environmental strains (*L. monocytogenes* 5404, P5326, P5642, PSU5402, and PSU5403). The clinical and environmental strains were kindly provided by associate professor Dr. Pimonsri Mittraparp-arthorn. The stock cultures were stored in 20% glycerol at −80 °C. The bacteria were cultured on a Tryptic Soy Agar (TSA) (37 °C, 18 h) and activated in Tryptic Soy Broth (TSB) under the same condition before antimicrobial testing.

### 2.3. Determination of Minimum Inhibitory Concentration (MIC) and Minimum Bactericidal Concentration (MBC)

The antimicrobial activity of COS and COS-PPN conjugates was measured by a modified colorimetric broth microdilution method [19]. COS and COS conjugated with catechin (COS-CAT), epigallocatechin gallate (COS-EGCG), gallic acid (COS-GAL), caffeic acid (COS-CAF), and ferulic acid (COS-FER) were diluted with the TSB medium by two-fold serial dilution in a 96-well microtiter plate for 10 dilutions to obtain 4 to 2048 µg/mL, and a volume of 50 µL was used. The inoculum of *L. monocytogenes* was prepared in the TSB medium to obtain 1 × 10^6^ CFU/mL, and 50 µL was then inoculated to each well, thus having final conjugate concentrations of 2–1024 µg/mL. The mixtures were subsequently incubated (37 °C, 15 h). The MIC was assessed by adding 10 µL of a resazurin dye solution (0.18%) to each well and incubated under the same condition for 3 h. The lowest concentration, which could inhibit bacterial growth, and caused no color change of resazurin (blue color), was considered as the ‘MIC’.

COS-PPN conjugates, which showed a higher concentration than the MIC and at the MIC, were selected for the MBC study. The MBC value was determined by the drop plate method on TSA plates, and the incubation took place at 37 °C for 18 h. The lowest concentration of COS or the COS-EGCG conjugate that killed bacteria (no growth) was considered as the ‘MBC’.

### 2.4. Time-Kill Assay

The time-killing of the COS-EGCG conjugate on *L. monocytogenes* was examined in the TSB medium by treating the bacterial cell (10^6^ CFU/mL) with the COS-EGCG conjugate at the MIC, 2 × MIC, and 4 × MIC. The bacterial culture treated with 0.01% acetic acid was named the negative control. The treatments were then incubated (37 °C, 24 h). During incubation, bacteria were counted at 0, 2, 4, 8, 12, and 24 h using a serial dilution with 0.85% normal saline solution (NSS), and the viable bacteria were counted on the TSA plates by the plate count method [20].

### 2.5. Effect of COS-EGCG Conjugate on Extracellular Polysaccharide Production

The COS-EGCG conjugate was prepared with the TSB medium to obtain the various concentrations, which were between 64 µg/mL (MIC/16) and 8192 µg/mL (8 × MIC), in sterile glass tubes. Thereafter, the inoculum of *L. monocytogenes* F2365 (10^6^ CFU/mL) was added. The final concentrations of the COS-EGCG conjugate were prepared in the range of 32 µg/mL (MIC/32) to 2048 µg/mL (2 × MIC). The mixtures were subsequently incubated at 37 °C for 48 h under the static condition. After incubation, the supernatants were collected by centrifugation at 2410× *g* for 15 min. The extracellular polysaccharide (EPS) production was determined by the phenol sulfuric acid method [21]. Briefly, the EPSs in the supernatant were precipitated with three volumes of chilled absolute ethanol at 4 °C for 24 h. The pellets were collected by centrifugation under the same conditions as mentioned above. The pellets were dissolved in a mixture of water, phenol (5% *v*/*v*), and concentrated sulfuric acid (1:1:5, *v*/*v*/*v*) and incubated at room temperature for 20 min. The solution was transferred to a 96-well microtiter plate, and the OD_490_ was measured to quantify EPSs in the solution. The percentage of EPS inhibition was then calculated [22].

### 2.6. Prevention of Biofilm Formation and Biofilm Inactivation of L. monocytogenes by COS-EGCG Conjugate

The procedure of Shi et al. [23] was adopted for the determination of biofilm prevention. Briefly, the COS-EGCG conjugate was prepared with the TSB medium in 96-well microtiter plates. *L. monocytogenes* F2365 was selected for the biofilm formation inhibition study of the COS-EGCG. This strain had the highest biofilm production among all strains tested. It showed moderate biofilm production (0.14 < OD_570_ < 0.28) [24]. The inoculum of *L. monocytogenes* F2365 (10^6^ CFU/mL) was prepared in the same medium and added to each well. The COS-EGCG conjugate, ranging from 4 µg/mL (MIC/256) to 2048 µg/mL (2 × MIC), was added, with a total volume of 200 µL. The mixtures were subsequently incubated at 37 °C for 48 h under the static condition. The bacterial growth was measured at OD_600_ using a FLUOstar Omega microplate reader (BMG Labtech GmbH, Ortenberg, Germany), and the planktonic cells were then discarded. The microtiter plate was then washed with sterile distilled water 5 times and air-dried. Staining of the biofilm was performed using 0.1% of a crystal violet solution at 25 °C for 45 min. The microtiter plate was rinsed with sterile distilled water 5 times and air-dried. The dried stain microtiter plate was added with 200 µL of 95% ethanol and left for 10 min. OD_570_ was measured, and the biofilm content was expressed as OD_570_.

For pre-biofilm inactivation, *L. monocytogenes* F2365, at a concentration of 10^6^ CFU/mL, was prepared in the TSB medium and transferred into a 24-well tissue culture plate (1 mL/well). The plates were incubated at 37 °C for 48 h under the static condition to perform the biofilm. The planktonic cells were discarded and washed with the PBS (1 mL). The COS-EGCG conjugates at concentrations ranging from 4 µg/mL (MIC/256) to 2048 µg/mL (2 × MIC) were prepared in Mueller Hinton Broth (MHB) and transferred to the 24-well tissue culture plate containing the biofilm. The plates were incubated at 37 °C for 24 h. After incubation, the planktonic bacteria and biofilm were removed from each well and gently washed twice with the PBS. The sessile bacteria in the biofilms were determined by the plate count method on the TSA plates [25].

### 2.7. Protein Leakages of L. monocytogenes F2365 Cells Treated with COS-EGCG Conjugate

*Listeria monocytogenes* cells, at the exponential growth phase, were suspended in a PBS (1 × 10^6^ CFU/mL) containing the COS-EGCG conjugate at the MIC, 2 × MIC, and 4 × MIC. The mixtures were subsequently incubated at 37 °C for 4 h. The untreated bacteria were considered a negative control. The collection of the cell suspension was conducted at different times (0, 1, 2, and 4 h), followed by filtration using a 0.22 µm Millipore filter. The amount of protein leaked into the filtrates was determined by the Bradford method [23]. OD_595_ was read using a FLUOstar Omega microplate reader (BMG Labtech GmbH, Ortenberg, Germany). A bovine serum albumin solution (1.95–15.6 µg/mL) was used as the standard protein.

### 2.8. The Effect of COS-EGCG Conjugate on Bacterial Genomic DNA

The extraction of the genomic DNA of *L. monocytogenes* F2365 was carried out using a QIAamp^®^ DNA Mini Kit (QIAGEN Sciences, Germantown, MD, USA). The ratio of OD_260_/OD_280_ (1.8 ≤ OD_260_/OD_280_ ≤ 2.0) and optical density at 260 nm (OD_260_), respectively, was measured with the aid of a NanoDrop Lite Plus Spectrophotometer (Thermo Fisher Scientific, Wilmington, DE, USA). The genomic DNA was diluted with 10 mM of a Tris-HCl buffer (pH 7.2) to obtain a final DNA concentration of 40 µg/mL. Additionally, the COS-EGCG conjugate was diluted with the same buffer to gain the various concentrations (0, MIC, 2 × MIC, and 4 × MIC). The treated DNA with the COS-EGCG conjugate at various concentrations and untreated DNA were measured on 0.8% agarose gel [23]. The quantitative measurement of DNA bands was done using ImageJ software and calculated as the percentage of band intensity, compared with the band of untreated DNA.

### 2.9. Anti-Motility Testing

Swimming and swarming motilities were assayed [26]. *L. monocytogenes* F2365 with overnight cultivation (2 µL) was placed at the center of a swimming soft agar (0.3% agar, 10 g/L tryptone, 5 g/L NaCl) and a swarming (0.5% agar, 25 g/L LB, 0.5 g/L glucose) soft agar in the presence of the COS-EGCG conjugate at MIC/4, MIC/2, MIC. Acetic acid (0.01%) was used as a control. After incubation (37 °C, 24 h), the colony diameters were measured.

### 2.10. Scanning Electron Microscopic (SEM) Observation

*L. monocytogenes* F2365 was incubated in the TSB medium at 37 °C for 18 h. The inoculum was prepared with 0.85% NSS to obtain 1 × 10^6^ CFU/mL. Five-hundred µL of inoculum were treated with 500 µL of the COS-EGCG conjugate at 4 × MIC (4096 µg/mL) and incubated at 37 °C for 18 h. The negative control consisted of the inoculum treated with 0.01% acetic acid. After incubation, the culture was centrifuged at 3773.25× *g* for 10 min, and the cell pellet was resuspended with the PBS. The cell suspension was applied to cover a glass slide coated with poly-L-lysine and placed at room temperature to adhere for 20 min. The adherent cells on the cover glass were fixed with 500 µL of 2.5% glutaraldehyde in a 24-well plate for 2 h at room temperature. The 2.5% glutaraldehyde was discarded and gently washed with 0.1 M of a phosphate buffer (pH 7.2) for 3 times, followed by sterile distilled water for 3 times. The fixed cells were dehydrated with a series of ethanol (50–100%), followed by critical point drying. The samples were then sputter gold-coated for 1 min and visualized using an FEI Quanta 400 Scanning Electron Microscope (FEI Czech Republic, Brno, Czech Republic).

### 2.11. Statistical Analysis

The experiments were conducted in triplicate, and the results for each test were reported as the mean ± standard deviation (SD). The results were subjected to a one-way analysis of variance (ANOVA), and a least significant difference test was implemented for the mean comparison. *p* < 0.05 was considered a significant difference.

## 3. Results and Discussion

### 3.1. Antimicrobial Activity of COS and COS Conjugated with Different Phenolic Compounds against L. monocytogenes from Various Sources

The *L. monocytogenes* strain F2365 was used for the antimicrobial activity testing of the COS and COS conjugated with varying phenolic compounds. The COS inhibited the growth of *L. monocytogenes* strain F2365 with the MIC and MBC of 2048 and 2048 µg/mL, respectively, while EGCG showed the MIC and MBC of 1024 and 2048 µg/mL, respectively (Table 1). Among all the conjugates treated, the COS-EGCG conjugate showed the highest inhibitory activity with the MIC and MBC of 1024 and 1024 µg/mL. Both the COS and COS-EGCG conjugate had a bactericidal effect on *L. monocytogenes* F2365 (an MBC/MIC ratio ≤ 4). The COS from the squid pen conjugated with EGCG showed bactericidal effects on *V. parahaemolyticus* with the MBC/MIC ratio of 3.97, *Salmonella enterica* with the MBC/MIC ratio of 3, *Pseudomonas aeruginosa* with the MBC/MIC ratio of 2, and *L. monocytogenes* with the MBC/MIC ratio of 1 [27]. On the other hand, the COS-CAT conjugate showed a bacteriostatic effect on strain F2365, with a low MIC of 256 µg/mL, but obtained a high MBC of 2048 µg/mL (an MBC/MIC ratio > 4). In general, the COS-CAF, COS-GAL, and COS-FER conjugates had the same MIC and MBC (2048 µg/mL) (Table 1). The antimicrobial activity and solubility of the COS were generally enhanced after being conjugated with polyphenol [18,28]. Therefore, the COS-EGCG conjugate was selected for testing with the clinical and environmental strains of *L. monocytogenes*. The result showed that the MBC/MIC ratio of all the strains was two-time (a bactericidal effect), except for strain LM14, which showed sixteen-time (a bacteriostatic effect) (Table 2). The result suggested that the COS-EGCG conjugate had a high potential to serve as an antimicrobial agent against different strains of *L. monocytogenes* associated with the environment and clinic. Nisin has been used as a biopreservative in RTE foods to inhibit the growth of *L. monocytogenes*. Nisin showed MIC and MBC values of 6.25 mg/mL against *L. monocytogenes* (goat cheese was associated with an outbreak strain) [29].

### 3.2. Suppression of Extracellular Polysaccharide Production by COS-GECG Conjugate

**Figure 1:**
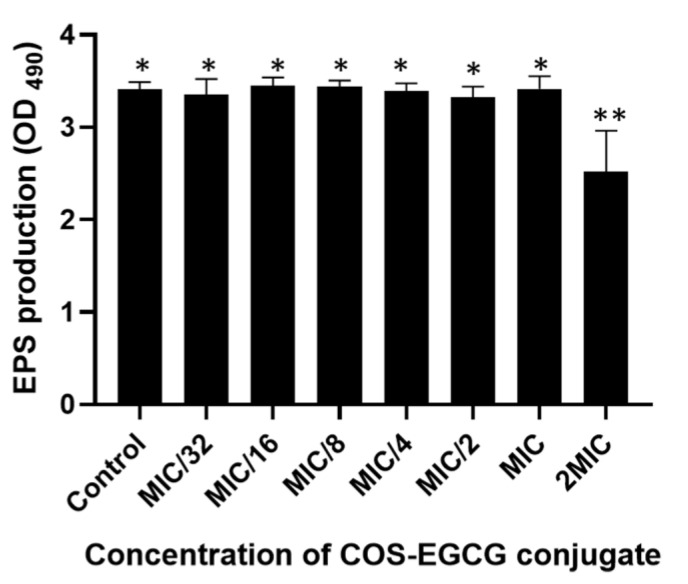
Extracellular polysaccharide production of *L. monocytogenes* F2365 treated with COS-EGCG at various concentrations in comparison with the control. The different asterisks on the bars denote significant differences (*p* < 0.05). The results are presented as means ± SD (*n* = 3).

EPSs are synthesized and secreted by microorganisms containing both polysaccharides (40–95%) and proteins (1–60%) [30]. EPSs can facilitate the initial adhesion of bacteria to different surfaces and trap the nutrients important for the development of biofilms in the early stage [31]. The matrix components of EPSs can encapsulate the bacterial cells to protect themselves from antimicrobial agents [32] and provide their tolerance to disinfectants and desiccation [33]. In the present study, the COS-EGCG conjugate at a concentration of 2 × MIC could reduce the EPS production by 26.1%, compared with the control. Nevertheless, the inhibition of EPS production by the COS-EGCG at concentrations from MIC/32 to MIC was not significantly different in comparison with the control (Figure 1). Therefore, the inhibition of EPSs by the COS-EGCG conjugate at a concentration of 2 × MIC markedly affected the reduction of the initial biofilm formation of the *L. monocytogenes*.

### 3.3. The Prevention and Inactivation of Biofilm by COS-EGCG Conjugate

**Figure 2:**
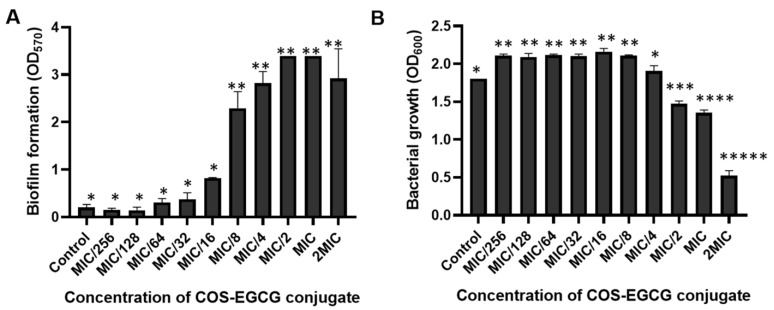
Biofilm formation (**A**) and the growth (**B**) of *L. monocytogenes* after treatment with COS-EGCG conjugate at different concentrations, as well as the control with 0.01% acetic acid in the TSB medium. The different asterisks on the bars denote significant differences (*p* < 0.05). The results are presented as means ± SD (*n* = 3).

*L. monocytogenes* has defense mechanisms against antibiotics, disinfectants, and environmental stresses via the formation of a biofilm [34,35]. Biofilms are formed on several surfaces of the food industry, such as utensils, instruments, etc. [36,37]. The biofilm formation of *L. monocytogenes* F2365 was examined after being treated with the COS-EGCG conjugate at various concentrations for 48 h. The biofilm formation increased when treated with *L. monocytogenes* F2365 with a high concentration of the COS-EGCG conjugate (from the MIC/8 to 2 × MIC). A significant difference (*p* < 0.05) was attained in comparison with lower concentrations and the control (Figure 2A). The low molecular weight COS (1–3 kDa) at a high concentration (512 µg/mL) can induce the formation of the biofilm of *L. monocytogenes* in a 96-well microtiter plate [34]. Low concentrations of antibacterial agents (less than the MIC or MBC) can cause bacterial injury. As a consequence, biofilms can be formed to protect themselves and survive in extreme conditions. The growth of *L. monocytogenes* treated with the COS-EGCG conjugate under the same condition with a biofilm formation assay was also determined. Overall, the growth was stimulated by the COS-EGCG conjugate at low concentrations (MIC/256 to MIC/8). However, the growth was suppressed when treated with the COS-EGCG conjugate at high concentrations (MIC/2, MIC, and 2 × MIC) compared to the control (Figure 2B). The COS-EGCG conjugate might be used as a carbon source at low concentrations by *L. monocytogenes* [34], whereas the growth was inhibited at higher concentrations as a result of cell disruption of *L. monocytogenes* by the COS-EGCG conjugate.

**Figure 3:**
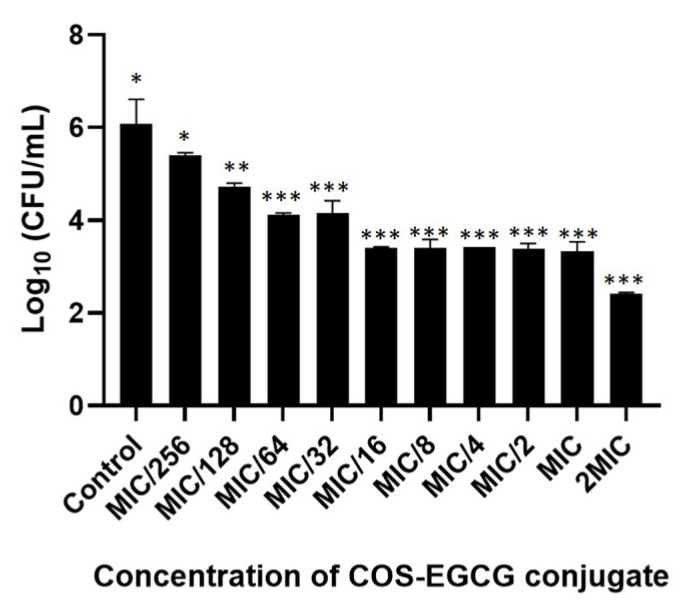
Inactivation of the biofilm of *L. monocytogenes* after treatment with COS-EGCG conjugate at different concentrations in comparison with the control. The different asterisks on the bars denote significant differences (*p* < 0.05). The results are presented as means ± SD (*n* = 3).

The inactivation of the biofilm was determined after 48 h of the biofilm formation in a 96-well microtiter plate. The COS-EGCG conjugates at different concentrations (the MIC, 2 × MIC, and 4 × MIC) diluted with MHB were applied to the well containing the biofilm to disrupt the pre-formed biofilm on the surface. The disrupted biofilms were removed by the PBS, and the sessile bacteria were counted on TSA plates. The sessile bacteria in the biofilms were inactivated by the COS-EGCG conjugate at concentrations ranging from MIC/128 to 2 × MIC. The COS-EGCG conjugate at a concentration of 2 × MIC could reduce the sessile *L. monocytogenes* F2365 by 3.7 log units, while the COS-EGCG ranging from MIC/128 to MIC could reduce the sessile bacteria from 1.3 to 2.8 Log CFU/mL (Figure 3). This result indicated that the COS-EGCG conjugate was effective in destroying pre-formed biofilms on the surface. The inactivation of the pre-formed biofilms is crucial and useful in managing microbial contamination in food processing plants or food contact surfaces. Moreover, the prevention of biofilm formation and inactivation of pre-formed biofilms using chitosan nanoparticles (ChNPs) and epsilon poly-l-lysine conjugated with chitosan nanoparticles (ChNP-PLs) on *L. monocytogenes* have been reported. The treatment of both ChNPs and ChNP-PLs reduced the biofilm formation by 184,000-fold compared with the untreated sample. ChNP-PL was also shown to inactivate pre-formed biofilms [25].

### 3.4. Time-Kill Analysis

**Figure 4:**
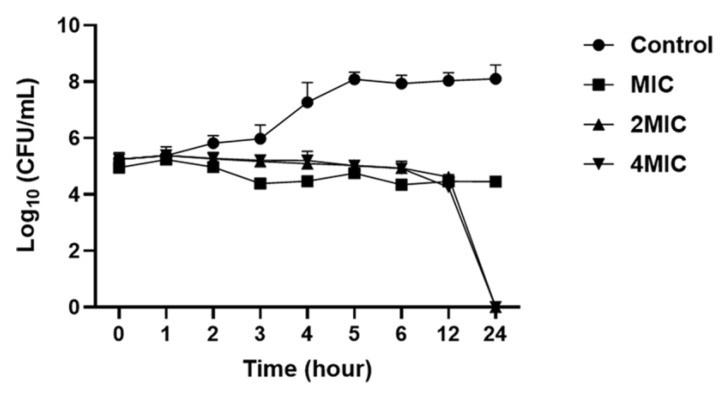
Time-kill curve of *L. monocytogenes* after treatment with COS-EGCG conjugate at different concentrations, as well as the control with 0.01% acetic acid in the TSB medium. The results are presented as means ± SD (*n* = 3).

The growth rate of *L. monocytogenes* rapidly decreased when treated with the COS-EGCG conjugate at 2 × MIC and 4 × MIC after 12 h, and the *L. monocytogenes* was undetectable after 24 h of treatment (Figure 4). Nonetheless, bacterial growth in the control was nearly 8.2 Log CFU/mL. Therefore, the COS-EGCG conjugate at a concentration higher than 2 × MIC had a bactericidal action on the *L. monocytogenes*. Although the COS-EGCG conjugate at a concentration of the MIC could not completely kill the *L. monocytogenes* after 24 h of treatment, the cells were reduced by almost 3 log units when compared to the control. Therefore, the COS-EGCG conjugate at the MIC could inhibit the growth of *L. monocytogenes* with bacteriostatic action. The time-kill assay of hexyl ferulate (FAC6) against *L. monocytogenes* was evidenced by the significant inhibition of bacterial growth and the occurrence of cell leakage. This compound also displayed both bacteriostatic and bactericidal effects on *L. monocytogenes* [23].

### 3.5. Protein Leakage

**Figure 5:**
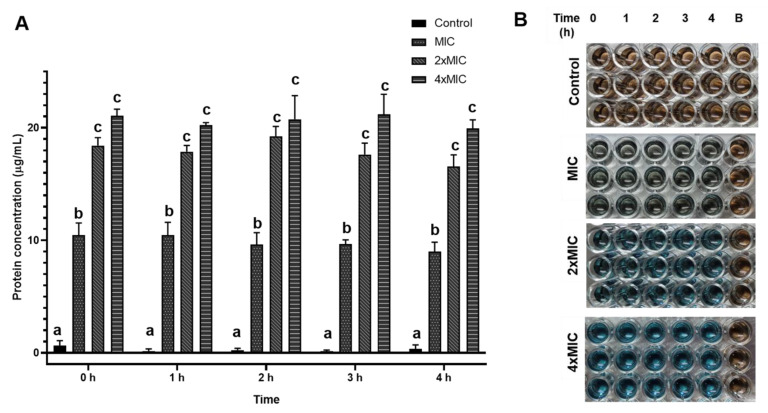
Quantitative (**A**) and qualitative (**B**) analyses of protein leakage of *L. monocytogenes* treated with COS-EGCG conjugate at various concentrations for different times. The different letters on the bars denote significant differences (*p* < 0.05). The results are presented as means ± SD (*n* = 3). The blue color indicates the presence of protein when tested using the Bradford assay.

The cell wall and cell membrane of bacteria are important structures to protect against cell damage by antibiotics, disinfectants, preservatives, and antibacterial substances [38,39]. The protein leakage from *L. monocytogenes* treated with the COS-EGCG conjugates at various concentrations for varying contact times was monitored (Figure 5). In general, the effectiveness of the COS-EGCG conjugates depended on the concentration, namely the “dose-dependent manner” (Figure 5A,B). The concentrations of protein that leaked from the *L. monocytogenes* cells treated with the COS-EGCG conjugate at 2 × MIC and 4 × MIC were not different (*p* > 0.05) but were higher than that of the MIC and the control (Figure 5A). In addition, the concentrations of protein that leaked from the *L. monocytogenes* cells in the treatments of 2 × MIC and 4 × MIC were nearly 19 µg/mL and 23 µg/mL, respectively. The leakage of protein from the cells treated with the MIC of the COS-EGCG conjugate was 10 µg/mL. *L. monocytogenes* cells treated with 4 × MIC of the COS-EGCG conjugate showed the highest protein leakage of approximately 23 times when compared to the control. It could be inferred that the COS-EGCG conjugate had a high effect on the gram-positive bacterial cell surface, resulting in the leakage of protein, and might cause the release of genetic materials or other cell constituents. However, the contact time had no profound impact on protein leakage. This was plausibly due to the rapid or sudden action of the COS-EGCG in interacting with the cell wall. As a result, a longer contact time showed no additional effect on protein leakage.

### 3.6. Effect of COS-EGCG Conjugate on DNA of L. monocytogenes

**Figure 6:**
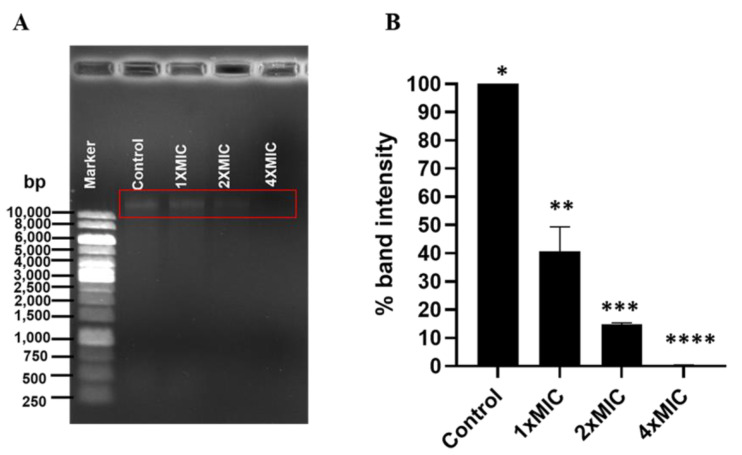
Agarose gel electrophoresis of genomic DNA of *L. monocytogenes* treated with COS-EGCG conjugate at different concentrations (**A**) and the band intensity (%) of DNA treated with COS-EGCG conjugate at various concentrations (**B**). The different asterisks on the bars denote significant differences (*p* < 0.05) The results are presented as means ± SD (*n* = 3).

DNA binding with antibacterial agents leads to bacterial cell death by the interference of gene expression, and it blocks the DNA polymerase. Those agents are also inserted into the DNA groove [23,40]. Besides the cell surface of *L. monocytogenes*, the genomic DNA was also bound with the COS-EGCG conjugates. The genomic DNA of the *L. monocytogenes* at a concentration of 40 µg/mL was treated with the COS-EGCG conjugate at the MIC, 2 × MIC, and 4 × MIC at 37 °C for 1 h. The intensity of the band decreased after being treated with the COS-EGCG conjugate at concentrations of the MIC and 2 × MIC when compared to that of the control (untreated) (Figure 6A). The percentage of the band intensity decreased to 40.7% and 14.8%, when the genomic DNA was treated with the COS-EGCG conjugate at the MIC and 2 × MIC, respectively (Figure 6B). The band of the DNA completely disappeared when treated with the COS-EGCG conjugate at 4 × MIC, mainly due to the formation of the COS-EGCG conjugate–DNA complex (Figure 6A). The effect of EGCG on the leakage of the cytoplasmic materials from the *Staphylococcus aureus* cell with the EGCG at 500 mg/L has been reported [41]. Moreover, EGCG at the same concentration reduced the growth of *S. aureus* by upregulating the expression of *gntP*, *gntK*, *rumA*, *SAOUHSC_02723*, *SAOUHSC_01311*, and *vraS* genes related to the membrane transport [41]. COS and chitosan with a low molecular weight and degree of polymerization (DP) can enter the bacterial cell and bind to the DNA, thus interfering with the DNA replication of bacteria [42]. The positive charge on the free amino group at C2 on the pyranose ring of the COS in the conjugate [18] might bind to the negative charge of the phosphate groups in the DNA structure. This was evidenced by a decrease in the band intensity of the genomic DNA on 0.8% of an agarose gel when treated with the COS-EGCG conjugate at concentrations of the MIC, 2 × MIC, and 4 × MIC. The DNA-binding rate of the COS-EGCG conjugate depended on the concentrations used. The 4 × MIC could bind to the DNA at a higher level than the 2 × MIC and MIC, respectively. The chitooligosaccharide-coated nanostructured lipidic nanoparticles at a 0.05–2% (*w*/*w*) concentration were completely complexed with the DNA (0.5 µg) [43]. The positive charges of the COS nanoparticles could bind to the negative charges of the phosphate groups in the DNA structure, resulting in the inhibition of DNA movement from the well [43].

### 3.7. Effect of COS-EGCG Conjugate on L. monocytogenes Motility

**Figure 7:**
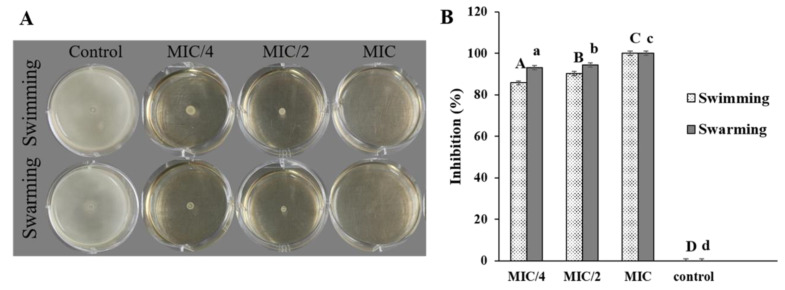
Effect of COS-EGCG conjugate at different concentrations on the mortality; swimming and swarming of *L. monocytogenes* MIC/4, MIC/2, and MIC, and sterile distilled water were used (**A**) and the percent inhibition of the motility of *L. monocytogenes* by the COS-EGCG conjugate was reported (**B**). Different uppercase letters and different lowercase letters on the bars for swimming and swarming, respectively, denote significant differences (*p* < 0.05).

**Figure 8:**
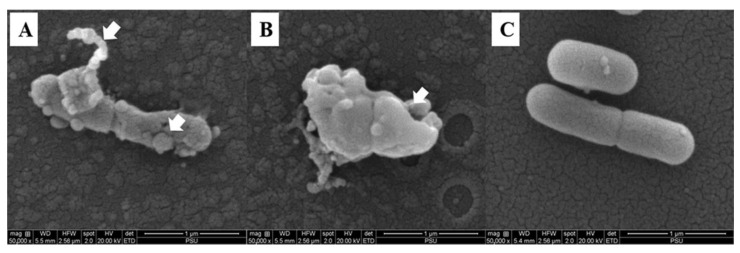
Scanning electron microscopic (SEM) images of *L. monocytogenes* treated with 4 × MIC of COS-EGCG conjugate (**A**,**B**) and treated with 0.01% acetic acid (negative control) (**C**). The magnification was 50,000×. Arrow signs indicate the distortion of flagella and rough surfaces (**A**) and cell surface damage with pores (**B**).

The effect of the COS-EGCG conjugate on *L. monocytogenes* motility, including swimming and swarming, is shown on the soft agar (Figure 7A). The COS-EGCG conjugate inhibited the swimming and swarming, with complete inhibition (100%) at the MIC, while MIC/4 and MIC/2 showed swimming and swarming inhibition in the range of 85.7–94.3% (Figure 7B). This result indicated that the motility inhibition was in a dose-dependent manner. All of the concentrations of COS-EGCG had a strong inhibitory effect on the motility of *L. monocytogenes*. Swimming and swarming are the important modes of bacteria movement to attach to biotic and abiotic surfaces for colonization and biofilm formation [44]. *L. monocytogenes* consists of 4–6 peritrichous flagella, which play a profound role in the movement. Basically, swimming is based on the movement of individual cells in liquid or semi-solid media using flagella, while swarming is the multicellular 2D movement over the tops of semi-solid surfaces using flagella [45]. The distortion of the flagella was found in *L. monocytogenes* treated with the COS-EGCG at a concentration of 4 × MIC (Figure 8A). Therefore, the COS-EGCG might lower the motility for the initial attachment of *L. monocytogenes* on food surfaces when its movement is impeded. However, some strains do not have flagella [46], or the flagella are inhibited by some selected agents [47,48]. As a result, the microorganisms cannot move. The antimicrobial agents were able to inhibit bacterial motility at the sub-MIC and higher.

### 3.8. Effect of COS-EGCG Conjugate on Morphological Changes of L. monocytogenes

The *L. monocytogenes* cells treated with 4 × MIC of the COS-EGCG conjugate showed cell distortion, a rough surface with degenerative changes, and cell wall damage with more pores on the cell surface (Figure 8A,B) when compared to the untreated cells (Figure 8C). This effect might be the cause of the leakage of nutrients and proteins from the bacterial cells. Moreover, the distortion of the filament was also found on the *L. monocytogenes* treated with the COS-EGCG conjugate (Figure 8A). The effect of EGCG with a concentration of 512 µg/mL on *Streptococcus suis* as gram-positive bacteria was reported [49]. The *S. suis* membrane was disrupted by the EGCG. Most of the cells lost their shape, resulting in the leakage of cytoplasmic content [49]. Generally, the gram-positive bacterial cell wall consists of several layers of peptidoglycan, which play a vital role in osmotic protection, cell division, and cell wall biosynthesis [50,51]. Moreover, COS has a high potential for bacterial cell wall disruption and leads to cell death [14,27]. COS has a mainly protonated form known as ammonium ion (-NH^3+^), which is positively charged and is able to bind with the peptidoglycan layer of bacteria containing teichoic acids (negatively charged) [52].

The antibacterial activity of the EGCG might be attributed to the -OH groups, which bind to the positively charged lipoproteins of the bacterial cell wall [53]. EGCG, as a phenolic compound, contains eight hydroxyl (-OH) groups. After being conjugated with COS, some free -OH groups were still available [18]. Thus, the morphological change of the *L. monocytogenes* could be induced by the COS-EGCG conjugate, mainly via the disruption of the *L. monocytogenes* cell surface, mainly via the destruction of the peptidoglycans on the cell wall of the gram-positive bacteria. Thus, both the COS and EGCG in the conjugate more likely worked in combination to disrupt the cell wall of the *L. monocytogenes* cells.

## 4. Conclusions

The antibacterial activity of COSs and different COS-PPN conjugates against *L. monocytogenes* indicated that the COS-EGCG conjugate showed the most efficacy in the inhibition of *L. monocytogenes* with bactericidal activity. The COS-EGCG conjugate at concentrations of 2 × MIC and 4 × MIC could completely kill *L. monocytogenes* after 24 h of incubation. The mechanisms of the COS-EGCG conjugate on the *L. monocytogenes* cells were the combined destruction of the cell surface and cell wall. The COS-EGCG conjugate brought about the leakage of protein from the cell and could also efficiently bind to the DNA of the *L. monocytogenes* at a concentration of 4 × MIC. In addition, the motility of *L. monocytogenes* was suppressed by a high level of the COS-EGCG conjugate, and no longer motility was detected at the MIC of the COS-EGCG conjugate. Moreover, the COS-EGCG conjugate at 2 × MIC had a high potential to inactivate the pre-biofilm, and it reduced the EPS production of the *L. monocytogenes*. Therefore, the COS-EGCG conjugate could be a potential antimicrobial agent in raw or ready-to-eat foods to control *L. monocytogenes*. The quantitative analysis of the DNA interaction with the COS-EGCG, molecular docking to predict the binding site, and gene expression using a DNA microarray will be investigated in the future.

## Figures and Tables

**Table 1:** Antibacterial activity of chitooligosaccharide (COS) and COS-polyphenol (PPN) conjugates (COS-PPN) against the reference strain of *L. monocytogenes* F2365.

Substances	MIC (µg/mL)	MBC (µg/mL)	MBC/MIC Ratio
COS	2048	2048	1
EGCG	1024	2048	2
COS-CAT	256	2048	8
COS-GAL	2048	2048	1
COS-CAF	2048	2048	1
COS-FER	2048	2048	1
COS-EGCG	1024	1024	1

COS: chitooligosaccharide; CAT: catechin; GAL: gallic acid; CAF: caffeic acid; FER: ferulic acid; EGCG: epigallocatechin gallate; MIC: minimum inhibitory concentration; MBC: minimum bactericidal concentration.

**Table 2:** Minimum inhibitory concentration (MIC) and minimum bactericidal concentration (MBC) of COS-EGCG conjugate against environmental and clinical strains of *L. monocytogenes*.

*L. monocytogenes* Strains	MIC (µg/mL)	MBC (µg/mL)	MBC/MIC Ratio
LM2	1024	2048	2
LM3	1024	2048	2
LM9	1024	2048	2
LM13	1024	2048	2
LM14	128	2048	16
EN5203	1024	2048	2
EN5326	1024	2048	2
EN5402	1024	2048	2
EN5404	1024	2048	2
EN5642	1024	2048	2

## Data Availability

The datasets generated for this study are available on request to the corresponding author.
